# Supplementary material for: Dual-Mode Aptamer AP1-F Achieves Molecular–Morphological Precision in Cancer Diagnostics via Membrane NCL Targeting
Source: Curr Issues Mol Biol. 2025 Oct 30;47(11):904. doi: 10.3390/cimb47110904 (PMC12650828; doi:10.3390/cimb47110904)
Supplement: Supplementary file 1 [file cimb-47-00904-s001.zip › cimb-3920121-supplementary.pdf]

current issues in molecular biology

Article

Dual-Mode Aptamer AP1-F Achieves Molecular–Morphological Precision in Cancer Diagnostics via Membrane NCL Targeting

Zhenglin Yang [1,2,†], Lingwei Wang [1,2,†], Chaoda Xiao [1,2,*] and Xiangchun Shen [1,2,*]

1 The High Efficacy Application of Natural Medicinal Resources Engineering Center of Guizhou Province, The High Educational Key Laboratory of Guizhou Province for Natural Medicinal Pharmacology and Drug Ability, School of Pharmaceutical Sciences, Guizhou Medical University, Guiyang 561113, China; crazyyang168@163.com (Z.Y.); w18984136703@163.com (L.W.)
2 The State Key Laboratory of Functions and Applications of Medicinal Plants, Guizhou Medical University, Guiyang 561113, China
* Correspondence: xcd@gmc.edu.cn (C.X.); sxc@gmc.edu.cn (X.S.); Tel./Fax: +86-085188416084 (C.X.); +86-085188416078 (X.S.)
† These authors contributed equally to this work.

Supplementary Materials

List of captions

Table S1. G4s sequence information used in this study.
Table S2. MST analysis fitting values.
Table S3. Experimental levels associated with each variable.
Table S4. Orthogonal test table and fluorescence intensity of corresponding cancer cells to normal cells in each group.
Table S5. Polar analysis values.
Table S6. Multifactor ANOVA analysis values.
Figure S1. Selective analysis of AS1411-F.
Figure S2. Mut-F staining of MCF-7, MDA-MB-468 and LX-2.
Figure S3. Laser confocal imaging results of aptamer Mut-F staining.
Figure S4. The cytotoxicity assay of AP1 and AS1411.
Figure S5. Mut-F staining of xenograft breast cancer tissues.
Figure S6. Anti-NCL Immunofluorescence Staining of Pathological Breast Cancer Tissue.
Figure S7. Analysis of Cancer Cell Recognition Capability in Mixed Cell Populations Mediated by AP1-F-Induced Forced Endocytosis.

Table S1. G4s sequence information used in this study

Aptamer names	Sequence
AP1[1]	GUUAGGGUUUGGGAUUG
AP2[1]	GUUAGGGUUUUGGGAUUG
AP3[1]	GUUAGGGUUUAUGGGAUUG
AP4[1]	GUUAGGGUUUAUUGGGAUUG
AP5[1]	GUUAGGGUUUAUUUGGGAUUG
AP6[1]	GUUAGGGUUUAUUAUGGGAUUG
AS1411[2]	GGTGGTGGTGGTTGTGGTGGTGGTGG

Mutant Sequence[1]	GUUAGAGUUUGAGAUUG

1. AP1-AP6 and Mutant sequence are RNAs. 2. AS1411 is DNA.

Table S2. MST analysis fitting values

Projects	AP1-F	AP2-F	AP3-F	AP4-F	AP5-F	AP6-F
K_d (nM)	4.74×10^{7}	6.21×10^{7}	4.99×10^{7}	8.22×10^{7}	9.26×10^{7}	5.07×10^{7}
K_d Confidence (±)	4.77×10^{-8}	2.69×10^{-7}	9.55×10^{-8}	2.90×10^{-7}	1.40×10^{-7}	1.32×10^{-7}
Response Amplitude	36.30	37.71	22.99	20.23	17.68	32.14
Std. Error of Regression	2.21	1.96	1.82	2.77	1.93	1.91
Reduced χ^2	11.58	7.57	43.16	3.23	7.64	1.27
Signal to Noise	14.56	23.96	13.76	10.36	13.34	18.46

Table S3. Experimental levels associated with each variable.

Levels	Centrifugation speed (×rpm)	Washing cycles	Centrifugation duration (min)	Staining temperature （℃）
1	1000	1×	5 min	4 ℃
2	2000	2×	10 min	25 ℃
3	3000	3×	15 min	37 ℃

Table S4. Orthogonal test table and fluorescence intensity of corresponding cancer cells to normal cells in each group.

Number	Centrifugation speed	Washing cycles	Centrifugation duration	Staining temperature	Mean ± SD
1	1	1	1	1	7.06±1.07
2	1	2	3	2	10.33±1.68
3	1	3	2	3	12.64±3.72
4	2	1	3	3	7.04±1.66
5	2	2	2	1	8.45±0.39
6	2	3	1	2	10.74±1.4
7	3	1	2	2	7.16±1.9
8	3	2	1	3	12.45±2.75
9	3	3	3	1	12.85±0.85
10	1	2	1	2	10.15±0.47

Table S5. Polar analysis values.

Project	Levels	Centrifugation speed	Washing cycles	Centrifugation duration	Staining temperature
	1	40.18	21.26	40.40	28.36
K value	2	26.24	41.38	28.26	38.38
	3	32.46	36.24	30.22	32.13
	1	10.05	7.09	10.10	9.45
K average value	2	8.75	10.34	9.42	9.60
	3	10.82	12.08	10.07	10.71
Optimal level		3	3	1	3
R		2.07	4.99	0.68	1.26
Number of levels		3	3	3	3
Number of replicates per level		3.0	3.0	3.0	3.0

Table S6. Multifactor ANOVA analysis values.

Projects	Square sum	df	Mean square	F	p
intercept	924.318	1	924.318	55465.597	0.003**
Centrifugation speed	6.536	2	3.268	196.093	0.050
Washing cycles	38.810	2	19.405	1164.434	0.021*
Centrifugation duration	0.853	2	0.427	25.602	0.138
Staining temperature	3.483	2	1.741	104.494	0.069
Residual	0.017	1	0.017		

R^2 = 1.000， *p<0.05， **p<0.01

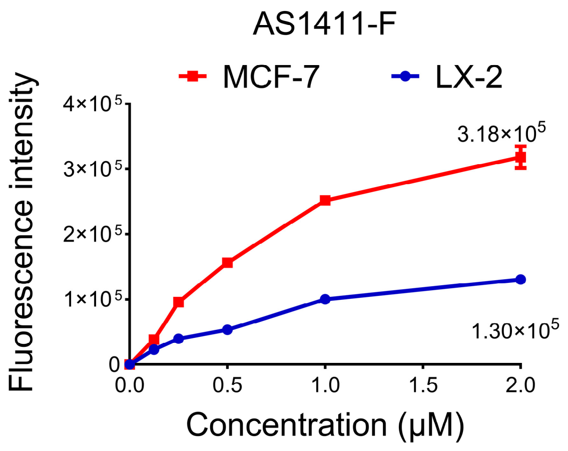

Figure S1. Variations in fluorescence intensity between MCF-7 cells and LX-2 normal cells at identical gradi-ent concentrations of AS1411-F staining circumstances (0 μM, 0.125 μM, 0.25 μM, 0.5 μM, 1.0 μM, 2.0 μM). Detection channel: FITC (fixed wavelength at λex/λem = 488 nm/530 nm). Data were expressed as the mean fluorescence intensity ± SD (n = 3).

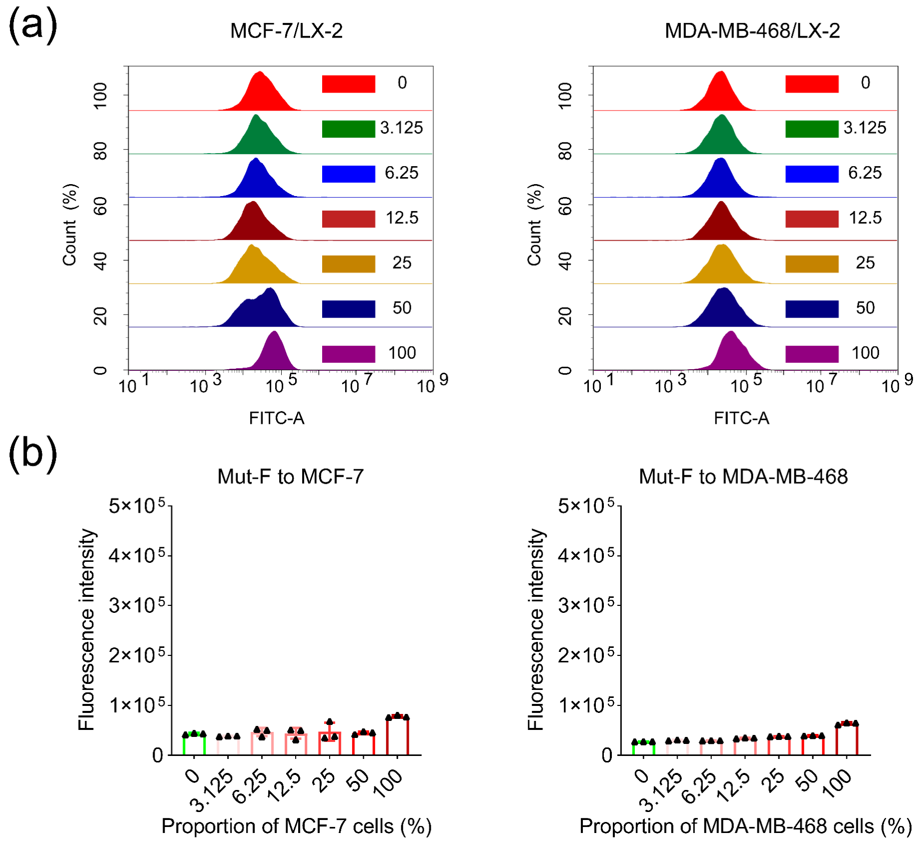

Figure S2. The study examines the effectiveness of Mutant-F in identifying target cells within a dual-cell mixed system (0%, 3.125%, 6.25%, 12.5%, 25%, 50%, 100%). (a) The flow cytometry results of mixed cell populations labeled with Mutant-F (1 μM). (b) Statistical analysis of fluorescence intensity in mixed cell samples stained with Mutant-F. Detection channel: FITC. The flow cytometry histogram findings were acquired at a constant wavelength of 488 nm/530 nm. Data were presented as the mean ± standard deviation (n = 3).

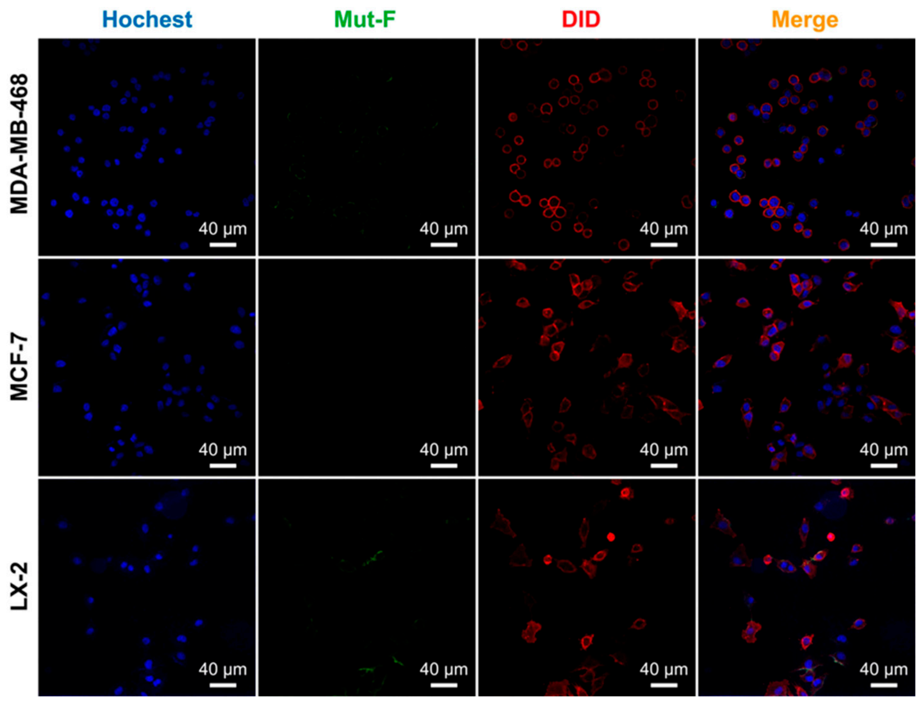

Figure S3. Laser confocal imaging results of aptamer Mut-F (1 μM) staining of MCF-7, MDA-MB-468 and LX-2. Green channel: λex = 488 nm, λem = 500–530 nm. Blue channel: λex/λem = 405 nm/440–470 nm. Red channel: λex/λem = 561 nm/600–700 nm. Scale bar: 40 μm.

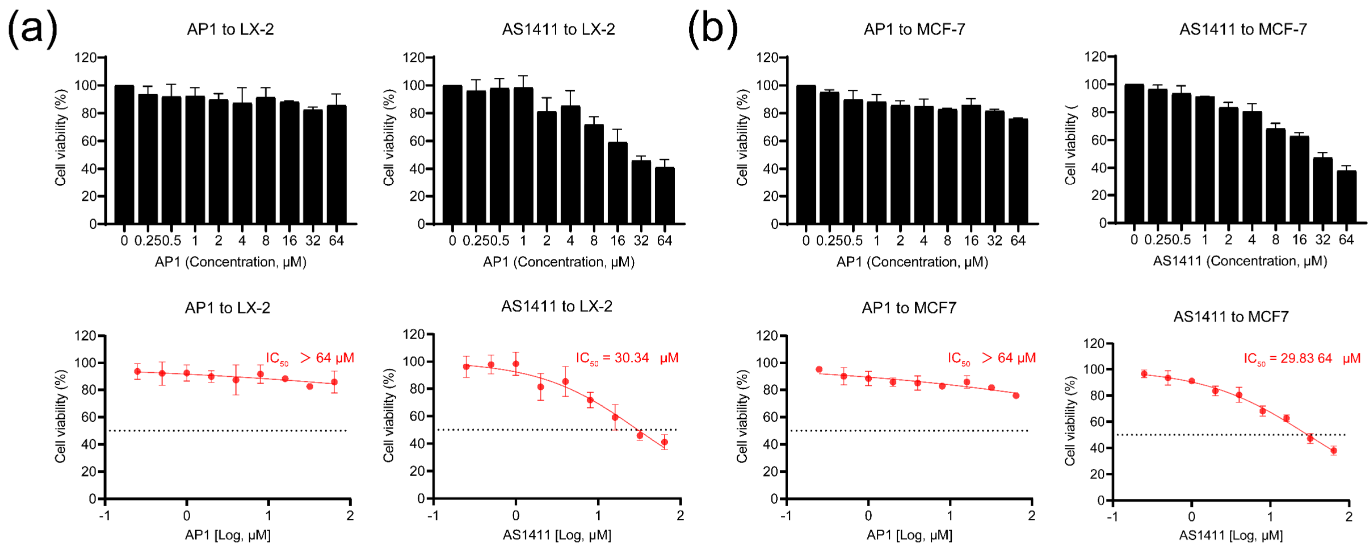

Figure S4. The cytotoxicity of AP1 and AS1411 was evaluated on normal cells LX-2 and breast cancer cells MCF-7, respectively. (a) To LX-2: AP1, IC50 > 64 μM; AS1411, IC50 = 30.34 μM; (b) To MCF-7: AP1, IC50 > 64 μM; AS1411, IC50 = 29.83 μM. Data were expressed as the mean ± SD (n = 3).

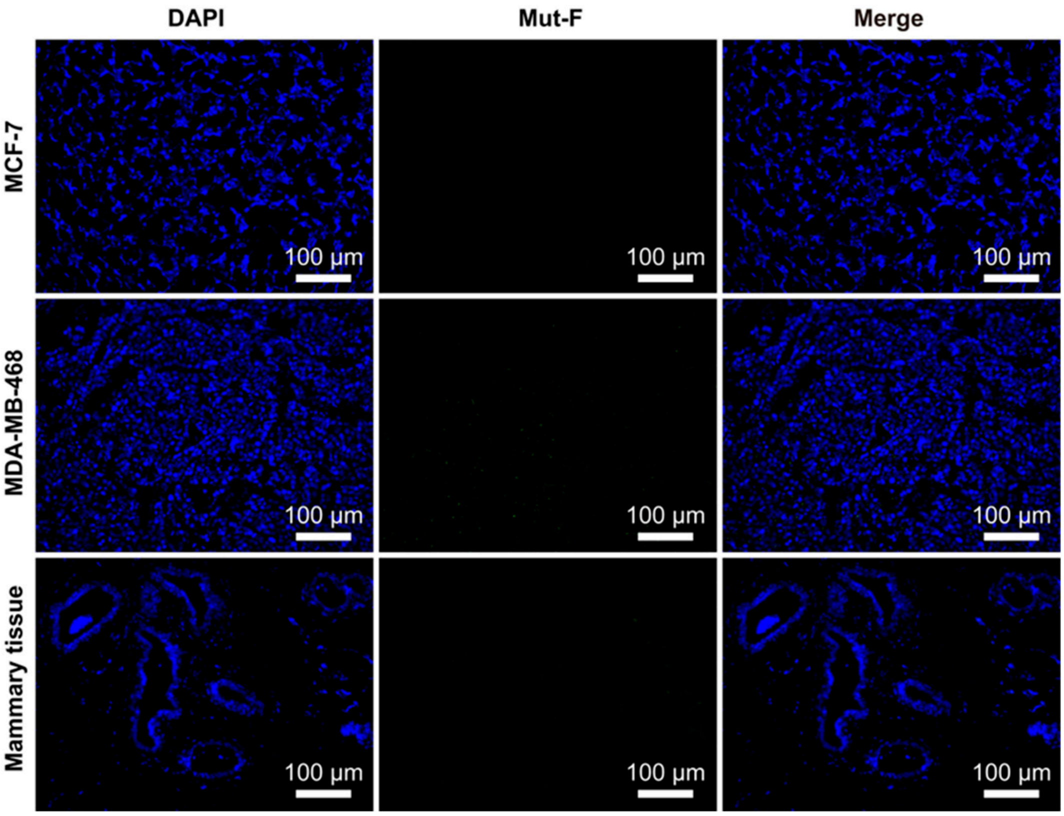

Figure S5. Mut-F (1 μM) staining of xenograft breast cancer tissues with two different molecular typologies (MCF-7 and MDA-MB-468) and Normal mouse mammary tissue. Green channel: λex/λem = 488 nm/500–530 nm. Blue channel: λex/λem = 405 nm/440–470 nm. Scale bar: 100 μm.

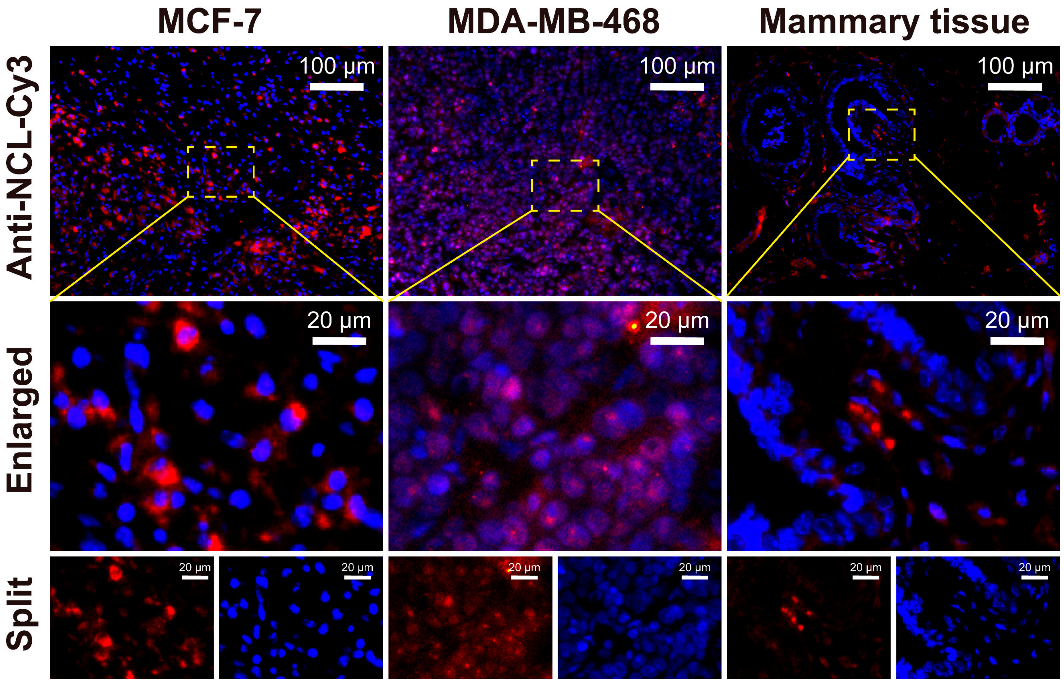

Figure S6. Immunofluorescence staining of pathologic tissue sections for breast cancer (MCF-7 and MDA-MB-468) and Normal mouse mammary tissue. Anti-NCL (1 μg/100 μL dilution) tissue immunofluorescence staining, Cy3 secondary antibody (1:400 dilution). Red channel: λex/λem = 561 nm/600–700 nm. Blue channel: λex/λem = 405 nm/440–470 nm. Scale bar: 20 μm.

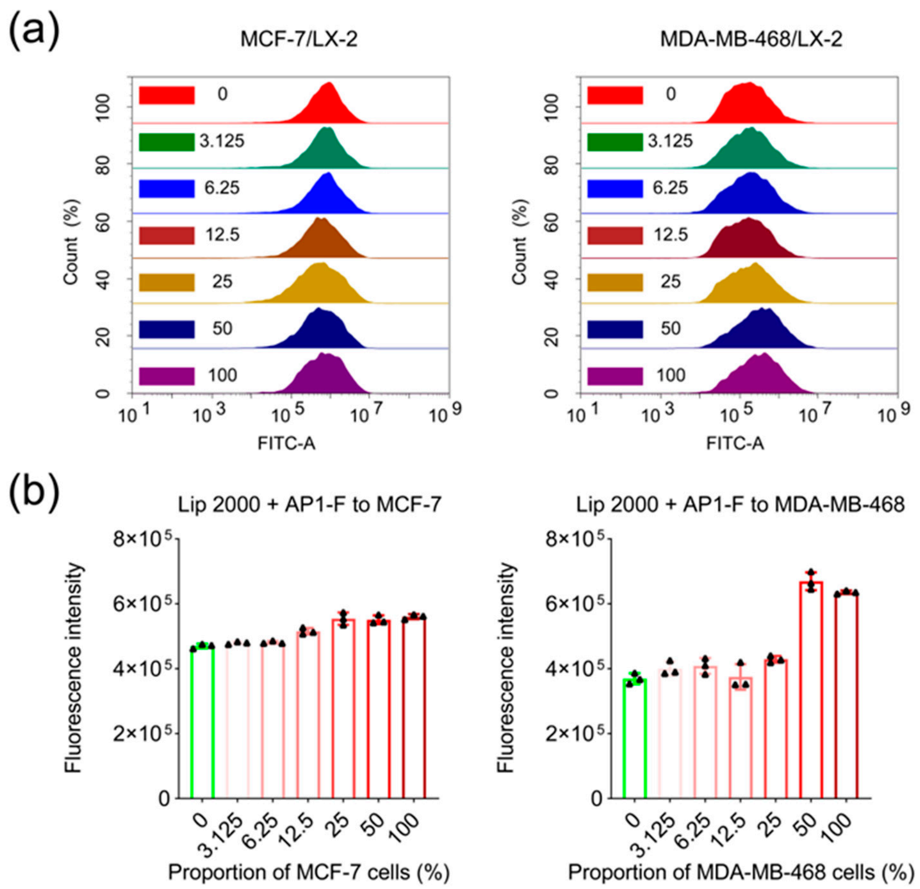

Figure S7. Analysis of Cancer Cell Recognition Capability in Mixed Cell Populations Mediated by AP1-F-Induced Forced Endocytosis. (a) The proportional mixed cell flow cytometry results of MCF-7/LX-2 and MDA-MB-468/LX-2. (b) The flow fluorescence intensity statistics of MCF-7/LX-2 and MDA-MB-468/LX-2 proportional mixed cells, respectively.
